# Association Between Serum Aluminum Level and Uremic Pruritus in Hemodialysis Patients

**DOI:** 10.1038/s41598-018-35217-6

**Published:** 2018-11-22

**Authors:** Ching-Wei Hsu, Cheng-Hao Weng, Ming-Jen Chan, Dan-Tzu Lin-Tan, Tzung-Hai Yen, Wen-Hung Huang

**Affiliations:** 1 Department of Nephrology and Clinical Poison Center, Chang Gung Memorial Hospital, Linkou Medical Center, Taoyuan, Taiwan; 2 Chang Gung University College of Medicine, Taoyuan, Taiwan

## Abstract

Uremic pruritus (UP) is a common symptom in patients undergoing hemodialysis (HD). The pathogenesis of UP is complex. Aluminum (Al) is a common metal and is toxic to patients undergoing HD. Al is also a known human allergen which can induce immune reactions. However, the correlation between Al and UP remains unclear in dialysis patients. A total of 866 patients on maintenance HD were enrolled for analysis. The HD patients with higher serum Al levels had higher a prevalence of UP than those with lower serum Al levels. After adjusting for confounding variables, the serum Al level was significantly associated with UP. Overall, each 10-fold increase in serum Al level was associated with a 5.64-fold increase in the risk of developing UP in these subjects. The results of this cross-sectional study suggest that serum Al level may be associated with the development of UP in patients on maintenance HD.

## Introduction

Uremic pruritus (UP) is a common and unpleasant symptom in patients with end-stage renal disease (ESRD). It impacts the quality of life and is associated with increased mortality in hemodialysis (HD) patients^1,2^. The prevalence of UP ranges from 42% to 90%^3,4^. Despite the high prevalence of UP, its pathogenesis is multi-factorial and poorly understood^1^. The main hypotheses of UP include the loss of normal skin function, inflammation, dysregulation of the endogenous opioidergic system, and central/peripheral neural systemic dysfunction^1^. Other factors that have also been implicated in the pathogenesis of UP include xerosis, increased parathyroid hormone, calcium phosphate-containing precipitates, iron deficiency anemia, hepatitis virus infection, and others^2^.

Aluminum (Al) is a toxic metal in humans, and its cumulative effects have been shown to be particularly detrimental to the health of ESRD patients^5^. An increased serum Al level has been associated with increased risks of dialysis dementia, renal osteodystrophy, anemia and mortality in patients undergoing maintenance HD^5,6^. The major sources of Al in maintenance HD patients are the water used for dialysate solution and Al-containing phosphate binders^5^. Since the 1980s, pretreatment of tap water by reverse osmosis and deionization has significantly reduced the Al concentration in dialysate solution^6,7^. The National Kidney Foundation–Kidney Disease Outcomes Quality Initiative suggests measuring the serum Al levels in maintenance HD patients at least once a year^8^. In 1997, Friga *et al*. demonstrated a positive correlation between serum Al levels and UP in 94 long-term HD patients^9^. However, few studies have investigated the association between Al and UP since Friga’s study. In particular, the association between serum Al level and UP is uncertain in maintenance HD patients. Therefore, we conducted this study to investigate the relationship between serum Al level and UP in this population.

## Results

### Patient characteristics

**Figure 1:**
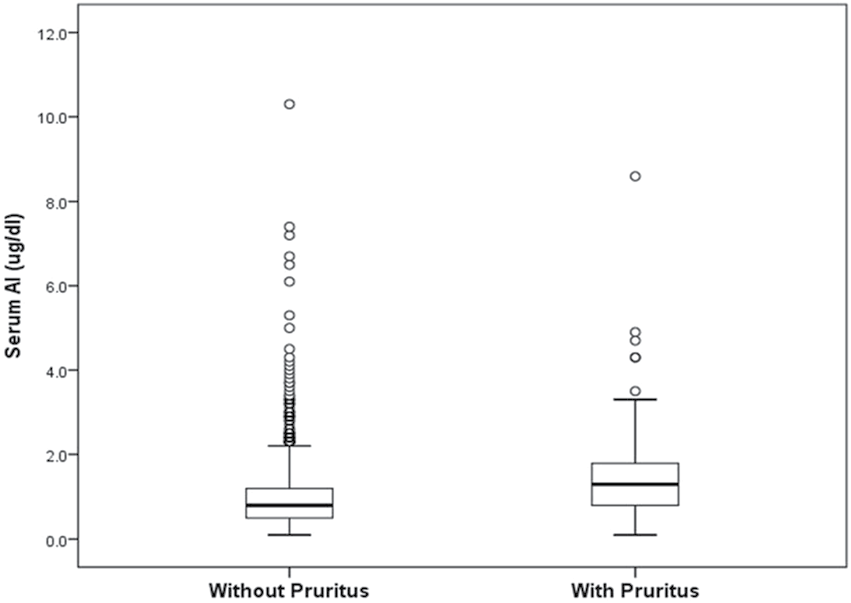
Box whisker plots shows the serum Al levels of patients without UP (n = 677) and with UP (n = 189). The whiskers indicate 95% confidence intervals, and dots represent outliers. The bottom and top of the boxes indicate 25 and 75 percentiles, respectively. The solid line within each box is the median. The serum Al levels in patients with UP was significantly higher than in patients without UP (*P* < 0.001). Data were compared using the Mann-Whitney U test. Abbreviation: Al, aluminum; UP, uremic pruritus.

A total of 866 maintenance HD patients (440 men and 426 women) met the study criteria and were included in this study. The mean age was 56.18 ± 13.59 years, and the mean HD duration was 6.96 ± 5.35 years. The median serum Al level was 0.9 ug/dL (range: 0.6–1.4 ug/dL). Among them, 189 patients (21.8%) had UP, 339 (39.1%) had hypertension, 192 (22.2%) had diabetic mellitus (DM), and 41 (4.7%) had previous cardiovascular diseases. As shown in Fig. 1, the serum Al levels in patients with UP (n = 189) was significantly higher than in patients without UP (n = 677) (*P* < 0.001). All patients were stratified into two groups based on the serum Al level: (1) serum Al level <2 ug/dL, n = 747; and (2) serum Al level ≥2 ug/dL, n = 119.

Table 1 shows the demographic and clinical characteristics, including comorbidities, dialysis-related information and biochemical data of the study patients overall and grouped by serum Al level. The patients with a higher Al level (≥2 ug/dL) had a higher prevalence of UP than those with a lower Al level (<2 ug/dL) (Table 1). The median numerical rating scale (NRS)^10^ score was 6 in the patients with UP. However, there was no significant correlation between serum Al level and NRS score by use of Spearman’s rank correlation test (r = 0.135, *P* = 0.064).

**Table 1:** Baseline characteristics of the study patients and comparisons of patients with different serum Al levels.

Characteristics	Total patients (n = 866)	Serum Al level <2 ug/dL (n = 747)	Serum Al level ≥2 ug/dL (n = 119)	*P*
***Demographics***				
Age (years)	56.18 ± 13.59	56.14 ± 13.69	56.49 ± 12.94	0.79
Male sex (Yes)	440 (50.8%)	50.9%	50.4%	0.99
Body mass index (kg/m^2^)	22.19 ± 3.18	22.16 ± 3.20	22.37 ± 3.05	0.50
Smoking (Yes)	150 (17.3%)	16.9%	20.2%	0.36
***Co-Morbidity***				
Hypertension (Yes)	339 (39.1%)	39.6%	36.1%	0.48
Diabetes mellitus (Yes)	192 (22.2%)	21%	29.4%	0.44
Previous cardiovascular disease (Yes)	41(4.7%)	4.8%	4.2%	0.99
Hepatitis B virus infection (Yes)	98 (11.3%)	11.1%	12.6%	0.64
Hepatitis C virus infection (Yes)	168 (19.4%)	18.3%	26.1%	0.60
Uremic pruritus (Yes)	189 (21.8%)	19.8%	34.5%	**0.001**
***Dialysis-Related Data***				
HD duration (years)	6.96 ± 5.35	6.84 ± 5.21	7.7 ± 6.14	0.10
Erythropoietin (U/kg/week)	73.62 ± 47.37	74.28 ± 47.23	69.44 ± 48.24	0.31
Fistula as blood access (Yes)	689 (79.6%)	79.1%	82.4%	0.46
Hemodiafiltration (Yes)	187 (21.6%)	21.8%	20.2%	0.81
Kt/V_urea_ Daugirdas	1.79 ± 0.32	1.80 ± 0.32	1.78 ± 0.32	0.51
Normalized protein catabolic rate (g/kg/day)	1.18 ± 0.26	1.18 ± 0.26	1.21 ± 0.29	0.18
Residual daily urine volume > 100 mL	178 (20.6%)	20.9%	18.5%	0.62
***Biochemical Data***				
Hemoglobin (g/dL)	10.51 ± 1.36	10.52 ± 1.34	10.47 ± 1.50	0.74
Albumin (g/dL)	4.06 ± 0.34	4.06 ± 0.34	4.03 ± 0.36	0.25
Creatinine (mg/dL)	10.88 ± 2.39	10.91 ± 2.38	10.71 ± 2.45	0.38
Ferritin (μg/L)	305.0 (129.57–504.45)	305.1(135.52–509.62)	305.7 (92.65–466.8)	0.13
Corrected-calcium (mg/dL)	9.94 ± 0.93	9.93 ± 0.90	10.01 ± 1.10	0.39
Phosphate (mg/dL)	4.84 ± 1.35	4.85 ± 1.34	4.78 ± 1.44	0.62
Intact parathyroid hormone (pg/mL)	130.1 (52.52–319.2)	127 (52.8–292.6)	152.9 (41.9–431.9)	0.20
High-sensitivity C-reactive protein (mg/L)	2.95 (1.4–7.01)	2.88 (1.37–7.01)	3.27 (1.57–7.01)	0.78
Serum Al (ug/dL)	0.9 (0.6–1.4)	0.8 (0.5–1.2)	2.5 (2.2–3.3)	**<0.001**
***Cardiovascular Risk Factors***				
Cholesterol (mg/dL)	171.3 ± 37.66	171.55 ± 37.11	169.76 ± 41.10	0.63
Triglyceride (mg/dL)	164.33 ± 115.8	163.54 ± 114.18	169.29 ± 125.93	0.61
Low density lipoprotein (mg/dL)	94.83 ± 30.59	95.27 ± 30.56	92.09 ± 30.81	0.29

Data are presented as mean ± standard deviation, number (%), or median (interquartile range) unless otherwise specified.

Abbreviations: Al, aluminum; HD, hemodialysis; Kt/V_urea_, dialysis clearance of urea.

### Associations among UP and serum Al levels and other clinical variables

In univariate logistic regression analysis, we identified 14 clinical variables that were associated with UP (Table 2), including body mass index, DM, hepatitis C virus infection, HD duration, hemodiafiltration, Kt/V_urea_, normalized protein catabolism rate, non-anuria status, serum albumin level, log intact parathyroid hormone, serum levels of cholesterol and low-density lipoprotein, log Al and serum Al level ≥2 ug/dL. After adjusting for the variables with *P* < 0.1 in univariate logistic regression analysis, log Al was significantly associated with a higher risk of UP (odds ratio = 5.64, 95% confidence interval = 3.13–10.17, *P* < 0.001) in multivariate logistic regression analysis with a forward method (Table 3). The chi-square of the Hosmer-Lemeshow goodness-of-fit test was 18.527 (*P* = 0.227).

**Table 2:** Univariate logistic regression analysis between uremic pruritus and clinical variables.

Variables	Odds ratio (95% confidence interval)	*P*
Body mass index (kg/m^2^)	1.07 (1.01–1.15)	0.036
Diabetes mellitus (Yes)	0.46 (0.29–0.73)	0.001
Hepatitis B virus infection (Yes)	0.56 (0.31–1.01)	0.058
Hepatitis C virus infection (Yes)	1.52 (1.03–2.23)	0.032
HD duration (years)	1.11 (1.05–1.15)	<0.001
Hemodiafiltration (Yes)	1.52 (1.05–2.20)	0.026
Kt/V_urea_ (Daugirdas)	2.94 (1.8–4.79)	<0.001
Normalized protein catabolic rate (g/kg/day)	1.93 (1.06–3.51)	0.03
Non-anuria	0.44 (0.27–0.71)	0.001
Albumin (g/dL)	0.57 (0.36–0.91)	0.02
Corrected-calcium (mg/dL)	1.18 (0.99–1.40)	0.056
Log ferritin	1.37 (0.96–1.95)	0.079
Log intact-parathyroid hormone	1.52 (1.14–2.02)	0.004
Cholesterol (mg/dL)	1.01 (1.00–1.01)	0.021
Low density lipoprotein (mg/dL)	1.01 (1.00–1.01)	0.005
Log Al	5.61 (3.27–9.61)	<0.001
Serum Al level ≥2 ug/dL (Yes)	2.12 (1.40–3.23)	<0.001

Variables including age, male sex, smoking status, hypertension, previous cardiovascular disease, fistula as blood access, hemoglobin, creatinine, phosphate, log high-sensitivity C-reactive protein and triglyceride had *P* values of >0.1 in univariate logistic regression analysis.

Abbreviations: Al, aluminum; HD, hemodialysis; Kt/Vurea, dialysis clearance of urea.

**Table 3:** Multivariate logistic regression analysis (forward method) between uremic pruritus and log Al level and other variables (with *P* < 0.1 in univariate logistic regression analysis).

Variables	Odds ratio (95% confidence interval)	*P*
HD duration (years)	1.09 (1.06–1.13)	<0.001
Diabetes mellitus	0.55 (0.33–0.91)	0.020
Non-anuria	0.58 (0.34–0.97)	0.038
Log ferritin	1.86 (1.26–2.74)	0.002
Low density lipoprotein (mg/dL)	1.01 (1.00–1.02)	0.001
Log Al	5.64 (3.13–10.17)	<0.001

Abbreviations:Al, aluminum; HD, hemodialysis.

In addition, after adjusting for the variables with *P* < 0.1 in univariate logistic regression analysis, a serum Al level ≥2 ug/dL was significantly associated with a higher risk of UP (odds ratio = 2.38, 95% confidence interval = 1.49–3.79, *P* < 0.001) in multivariate logistic regression analysis with a forward method (Table 4). The chi-square of the Hosmer-Lemeshow goodness-of-fit test was 19.479 (*P* = 0.180). Moreover, Fisher’s exact test demonstrated that the patients with a high serum Al level had a significantly higher incidence of UP than those with a low serum Al level (34.5% *vs*. 19.8%, *P* < 0.001) (Table 5).

**Table 4:** Multivariate logistic regression analysis (forward method) between uremic pruritus and serum Al level ≥2 ug/dL and other variables (with *P* < 0.1 in univariate logistic regression analysis).

Variables	Odds ratio (95% confidence interval)	*P*
HD duration (years)	1.11 (1.07–1.14)	<0.001
Diabetes mellitus	0.57 (0.34–0.93)	0.024
Albumin (g/dL)	0.56 (0.34–0.94)	0.027
Log ferritin	1.83 (1.24–2.69)	0.002
Low-density lipoprotein (mg/dL)	1.01 (1.00–1.02)	0.001
Serum Al level ≥2 ug/dL (Yes)	2.38 (1.49–3.80)	<0.001

Abbreviation: Al, aluminum; HD, hemodialysis.

**Table 5:** Fisher’s exact test of the prevalence of uremic pruritus by serum Al level.

Variables	Serum Al <2 ug/dL	Serum Al ≥2 ug/dL	Total patients	Odds ratio	*P*
Without pruritus	599 (80.2)	78 (65.5)	677	2.13	<0.001
With pruritus	148 (19.8)	41 (34.5)	189		
Total patients	747	119			

Data are presented as number (%).

Abbreviation: Al, aluminum.

## Discussion

The analytical results of this study demonstrated that the maintenance HD patients with a higher serum Al level had a higher prevalence of UP than those with a lower serum Al level. Following adjustments for confounding variables, the serum Al level was significantly associated with UP in our study patients with ESRD. Overall, each 10-fold increase in serum Al level was associated with a 5.64-fold increase in the risk of developing UP in these subjects.

The mechanisms of UP are complex and many hypotheses have been proposed for the possible underlying etiology, none of which have been conclusively proven. Of these, inflammation seems to be important in the pathogenesis of UP due to the release of certain cytokines during HD^11^. UP may also be induced by mast cell proliferation and increased deposits of calcium-phosphate complex in the skin due to secondary hyperparathyroidism^11^. In addition, abnormalities interactions between dermal mast cells and the distal ends of nonmyelinated C fibers may play a role in UP through the release of various triggering substances from mast cells, including histamine, proteases, interleukin-2, and tumor necrosis factor-α^12^. Several other factors have also been implicated in the pathogenesis of UP, including xerosis^2^, environmental air pollution^13^, inadequate dialysis^14^, serum phosphate^15^ and magnesium^16^, iron deficiency anemia^17^, neuropathy and neurological changes^18^, hepatitis virus infection and others^2^.

Few studies have investigated the association between serum levels of Al and pruritus in dialysis patients. A study of 94 HD patients by Friga *et al*.^9^ showed a positive relationship between serum Al level and pruritus, and the intensity of pruritus was significantly associated with the concentration of Al. However, such a correlation was not observed in another similar study by Carmichael *et al*.^15^ which included 54 HD patients. In the general population, persistent pruritus and itching nodules have been reported after the use of Al-containing vaccines^19–21^. Bergfors *et al*.^19^ reported a high incidence of pruritic nodules after the administration of diphtheria-tetanus/acellular pertussis vaccines in 77% of children associated with Al allergy. The mechanism between serum Al and pruritus in maintenance HD patients is unknown. Type 1 hypersensitivity reactions have been suggested to be a pathway inducing an allergic reaction^22^, which may play a role in pruritus in ESRD patients. We did not have information about which of our patients were allergic to Al, as no data on contact-hypersensitivity tests for Al were available. In this study, the prevalence rate of pruritus in the dialysis patients was 13.7%, which is much higher than the allergic rate to Al-containing vaccines of about 0.8% (645/76000) reported by Bergfors *et al*.^19^. This may be because UP is complex and multifactorial (the significant factors in this study were HD duration, anuria status, ferritin and low-density lipoprotein levels), and many unknown clinical variables may also exist. Further studies are needed to elucidate the mechanism of the association between UP and serum Al, and to determine whether HD patients are at a greater risk of Al-induced hypersensitivity than the general population.

In multivariate logistic regression analysis, the odds ratios of UP were 5.64 for log Al and 2.38 for serum Al level. Al-contaminated dialysate has not been an issue for maintenance HD patients due to the use of reverse osmosis and deionization techniques since the 1980s^7^. Hence, it is important for maintenance HD patients to reduce their exposure to Al in their daily life. For example, Al-based phosphate binders are still prescribed for ESRD patients to treat severe hyperphosphatemia^8^. The use of Al-containing antacids may also increase the serum Al levels in dialysis patients^6^. In addition, cooking with Al utensils may be a source of exposure in ESRD patients^5,23^. Furthermore, Al-containing food ingredients, including preservatives, coloring agents and anticaking agents, may be another source of Al exposure^24^. All of these possible sources of Al should be avoided for maintenance HD patients. However, further studies are needed to explore whether reducing serum Al levels can decrease the risk of UP in dialysis patients.

Urinary excretion is an important route for the elimination of toxic metals in humans. The metabolism of digested Al is characterized by low intestinal absorption, rapid urinary excretion, and slow tissue uptake, mostly in skeletal and reticuloendothelial cells^25^. This may explain why residual renal function played an important role in our patients with UP (Table 3). However, this correlation was not found in the other multivariate logistic regression analyses (Table 4). Further studies are needed to confirm these findings.

Keithi-Reddy *et al*.^26^ reported that DM was a possible cause of itching in ESRD patients. However, we found that DM was negatively associated with UP in the current study. Furthermore, we did not find a correlation between DM and UP in our previous report on the association between environmental air pollutants and UP^13^. It is known that diabetic patients are immunocompromised due to impaired phagocytic activity of mononuclear cells, and therefore are at an increased risk of infections^27^. Whether or not the decrease innate immunity in diabetic patients is negatively correlated with pruritus in HD patients is unknown. However, taken together, DM does not appear to be strongly correlated with UP.

Serum ferritin is a well-known inflammatory marker^28^. In this study, we found that serum ferritin levels were positively associated with UP in multivariate logistic regression analysis (Tables 3 and 4). In further analysis using Pearson’s correlation coefficient, log ferritin level was positively correlated with DM (r = 0.17, *P* < 0.001), Kt/V_urea_ (r = 0.13, *P* < 0.001), and non-anuria (r = 0.073, *P* = 0.032). It was also negatively correlated with body mass index (r = −0.07, *P* = 0.039), hepatitis B virus infection, (r = −0.068, *P* = 0.046), hepatitis C virus infection (r = −0.176, *P* < 0.001), duration of HD (r = −0.151, *P* < 0.001), use of hemodiafiltration (r = −0.118, *P* = 0.001), corrected calcium (r = −0.129, *P* < 0.001), and log intact parathyroid hormone level (r = −0.161, *P* < 0.001). After adjusting for these correlated variables, log ferritin level was positively correlated with UP, which is consistent with previous studies^12,13,29^. Taken together with our findings, inflammation may explain why these factors were correlated with UP.

There are several limitations to this study. First, this is a single-center and cross-sectional study. Even though we found a correlation between serum Al levels and UP, the number of patients was moderate and they were randomly selected. Nevertheless, we believe that the design of this study is objective and reasonable. Second, we did not have data on how many patients were allergic to Al. Therefore, we may have underestimated or overestimated the correlation between serum Al and UP. UP is difficult to treat, and there may be as yet unidentified factors associated with UP. Third, we did not have data on the Al content of the patients’ diet or drinking water. However, in this study we used serum Al levels rather than diet or drinking water for analysis. Therefore, the observation and analysis are objective.

In conclusion, the results of this cross-sectional study demonstrated that serum Al level was significantly associated with UP in the dialysis patients. Further studies are required to clarify the role of serum Al on UP in patients undergoing maintenance HD.

## Methods

### Ethics statement

This clinical study was conducted in accordance with the Declaration of Helsinki and was approved by the Institutional Review Board of Chang Gung Memorial Hospital, a tertiary referral center in Taiwan. Since this study involved the retrospective review of existing data, the Institutional Review Board specifically waived the need for written informed consent. The information for each patient was securely protected by delinking any identifying information from the main dataset. In addition, all data were only available to the investigators and were analyzed anonymously.

### Patients

**Figure 2:**
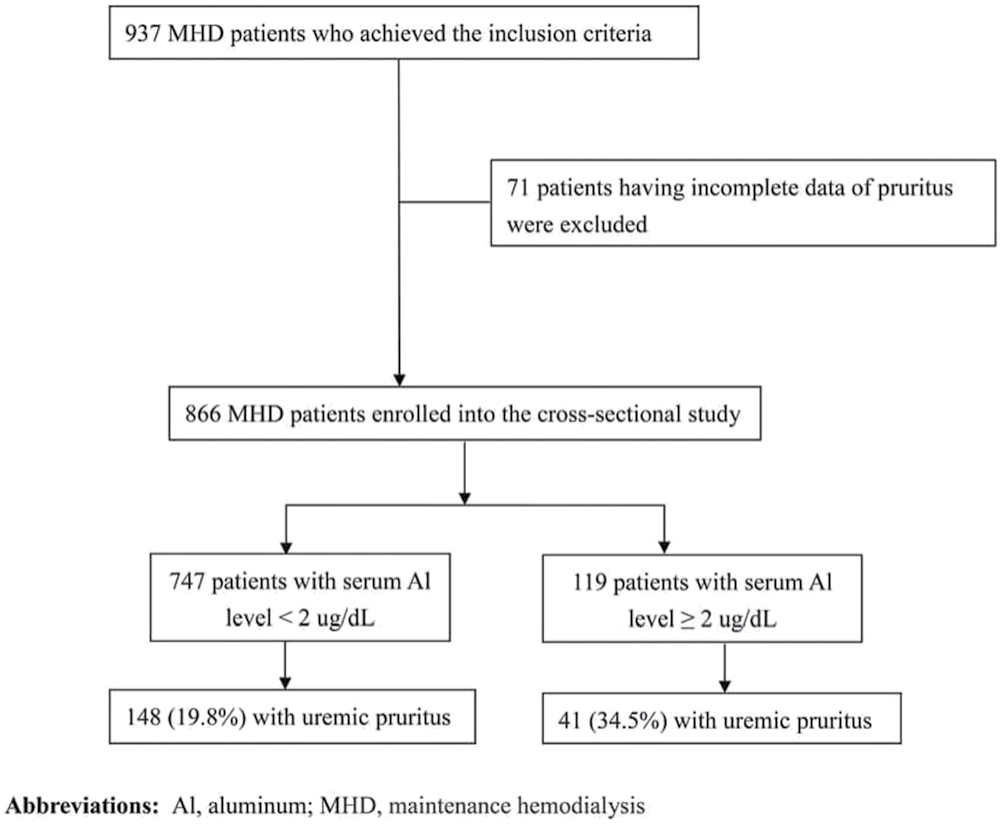
Flow chart of patient recruitment.

All patients were recruited from the three HD centers of Chang Gung Memorial Hospital (Taipei, Lin-Kou, and Taoyuan). Only maintenance HD patients who had undergone HD for at least 6 months, were aged ≥18 years, and had studies of serum Al levels were enrolled (Fig. 2). Patients with malignancies, obvious infectious diseases, or who had been hospitalized or undergone surgery in the 3 months before enrollment were excluded.

Most of the patients were treated with 4 h of HD three times per week. HD was conducted with single-use hollow-fiber dialyzers fitted with modified cellulose, polyamide or polysulfone membranes. The dialysate was a standard ionic composition with bicarbonate-based buffer, and a standard reverse osmosis system was used for water purification. Patients undergoing hemodiafiltration for more than 6 months were also included.

Patients with hypertension were defined those with blood pressure ≥140/90 mmHg based on at least two measurements or the regular use of antihypertensive drugs. Patients with DM were defined as those with a history of diabetes diagnosed by a physician or the presence of two measurements of fasting glucose >126 mg/dL. The prevalence of cardiovascular diseases, including cerebrovascular disease, coronary arterial disease, congestive heart failure and peripheral vascular disease, was recorded. Smoking status was also recorded.

The diagnosis of pruritus was defined as pruritus appearing after HD with or without antipruritics, as observed by trained dermatologists or nephrologists. Pruritus may be constant or intermittent and is commonly associated with xerosis. Pruritus commonly affects the arms, head, and abdomen, and the back is the most commonly affected area. We used a NRS to evaluate the intensity of pruritus in our study patients. The NRS is a single 11-point numeric scale. The patients were asked to assign a numerical score on the NRS that best represented the intensity of their symptoms on a scale from 0 to 10, with 0 referring to no pruritus and 10 to the worst imaginable symptoms.

### Measurement of serum Al levels

To ensure that the patients were not exposed to Al-contaminated water and dialysate during HD, we collected at least two samples of dialysate from the inlets and outlets of the dialysate part of the dialyzers at each HD center into Al-free plastic tubes. Blood samples were centrifuged to separate serum. All samples were deproteinized using trichloroacetic acid and microwave irradiation before measurements. All steps of sample preparation were conducted under a laminar flow hood. Al was measured by graphite furnace atomic absorption spectrometry using a Perkin-Elmer 5100 (Norwalk, CT, USA) atomic absorption spectrometer with Zeeman background correction and an L’vov platform equipped with a graphite furnace and an auto sampler. Distilled and de-ionized water was used throughout all procedures. An Al standard solution containing 1000 mg/L Al (Merck, Germany) was used to prepare the working standard solutions. Nitric acid (HNO_3_, 65% m/m, 1.17 g/mL; Merck, Germany) was further purified by sub-boiling distillation. Only plastic materials were used to avoid contamination. All laboratory ware (pipette tips, volumetric flasks, etc.) were immersed for at least 48 h in a 10% (v/v) HNO_3_/ethanol solution and washed with purified water shortly before use. To avoid contamination from the air, all steps of the sample and reagent preparation were carried out in a clean bench. This study used both internal and external quality-control procedures, and the results were consistently satisfactory. A certified commercially prepared product (Seronorm Trace Elements, Sero AS, Billingstads, Norway) was used to determine intra-batch accuracy and to ensure inter-batch standardization. The intra- and inter-batch coefficients of variation for the Al measurements were ≤5.0%. The detection limit was 0.1 μg/L. External quality control was maintained via participation in the National Quality Control Program conducted by the government.

### Laboratory, nutritional, and inflammatory parameters

Blood samples of the study patients were obtained during stable HD sessions to minimize the influence of acute events. All samples were taken from the arterial end of the vascular access immediately before the start of the mid-week HD session. The samples were then centrifuged and stored at −80 °C until analysis.

Serum levels of albumin and creatinine and normalized protein catabolism rate were assayed and recorded as nutritional markers. High-sensitivity C-reactive protein was used as a marker of inflammation and measured via immunonephelometry (Nanopia CRP; Daiichi, Inc., Tokyo, Japan), with the lowest detection limit being <0.15 mg/L. The normalized protein catabolism rate was calculated using validated equations and normalized to actual body weight^30^. All other biochemical parameters were measured using an automatic chemistry analyzer via standard laboratory approaches. Dialysis clearance of the urea was expressed as Kt/V_urea_, using the method described by Daugirdas^31^ for dialysis patients. Serum calcium level was corrected for serum albumin level using the following formula: corrected calcium (mg/dL) = serum calcium (mg/dL) + 0.8 × (4.0 − serum albumin [g/dL]). Anuria was defined as a daily urine volume of <100 mL. A serum Al level <2 ug/dL was considered as being unlikely to cause Al toxicity^8^.

### Statistical analysis

The Kolmogorov-Smirnov test was used to test whether the variables were normally distributed, and a *P* value > 0.05 was considered to indicate normal distribution. Continuous variables were expressed as means ± standard deviations or medians with interquartile ranges, and categorical variables were expressed as numbers with percentages. We used the χ^2^ test or Fisher’s exact test to compare categorical variables, and the Student’s *t*-test or Mann-Whitney *U* test to detect significant differences between study groups. Logarithmic conversion was conducted for variables without normal distribution, including high-sensitivity C-reactive protein, intact parathyroid hormone, ferritin and serum Al levels.

To evaluate the variables associated with UP, we used univariate and multivariate logistic regression model to determine the odds ratio and 95% confidence interval of the clinical variables. All potential variables (*P* < 0.1) in univariate logistic regression analysis were entered into multivariate logistic regression models with forward methods. We also used the Hosmer-Lemeshow test to assess goodness-of-fit of this model.

The data were analyzed using the Statistical Package for the Social Sciences (SPSS) version 18.0 for Windows 7 (SPSS Inc., Chicago, IL, USA). A *P* value of < 0.05 was considered statistically significant.
